# Nutrition Promotion for Children in Schools: A Content Analysis of Nutrition Marketing in Arizona School Cafeterias

**DOI:** 10.21203/rs.3.rs-9139962/v1

**Published:** 2026-04-17

**Authors:** Mary Curnutte, Raevyn Xavier, Eden Skaer, Cori Lorts, Marc Adams, Meg Bruening

**Affiliations:** Pennsylvania State University; Arizona State University; Pennsylvania State University; Northern Arizona University; Arizona State University; Pennsylvania State University

**Keywords:** school nutrition, nutrition marketing, school lunch, child nutrition, national school lunch program

## Abstract

**Background:**

Children consistently fall short of the recommended intake of fruits and vegetables while consuming excess nutrient-poor foods, which is associated with adverse health outcomes and academic performance. Food and beverage companies widely promote nutrient-poor foods to children. Schools reach tens of millions of students daily, providing an opportunity to market healthful choices, yet few studies have examined effective healthful nutrition marketing in schools. This study examined the content of nutrition marketing materials within elementary, middle, and high school cafeterias in Arizona to understand the current strategies being used to promote nutrition to students.

**Methods:**

Photographs of existing nutrition marketing within 37 school cafeterias in Arizona, including elementary (n = 13), middle (n = 12), and high schools (n = 12) between 2017–2020, were taken, and an analysis was conducted to code for content, purpose, prevalence, visual messaging, and literary messaging used to influence eating behaviors.

**Results:**

The most common marketing material was posters, the most common behavior target was selecting a fruit or vegetable, the most common content was social media or website links followed by the use of cartoon characters, the most common messaging strategy was gain-frame (framing a message by highlighting the benefits of engaging in a behavior), the most common outcome was short-term messages, and the reading level was not appropriate for most elementary and middle schools.

**Conclusions:**

Some, but not all, nutrition marketing in the sampled schools mirrors current effective food marketing. This research can serve as a baseline of current nutrition marketing strategies to improve nutrition marketing for students.

## 1.1 Introduction

Children in the U.S. consistently fall short of recommended fruit and vegetable intake while consuming excess energy-dense and nutrient-poor foods (Hamner, 2023). This dietary pattern is associated with adverse developmental outcomes, such as increased chronic disease risk (Sahoo et al., 2015; Van der Aa et al., 2015), increased risk of mortality (Fruit and Vegetable Consumption Reduce Risk of Death / National Institutes of Health (NIH), n.d.), greater rates of anxiety and depression (Głąbska et al., 2020), and lower academic performance (Alaimo et al., 2001; Bellisle, 2004; Florence et al., 2008; Glewwe et al., 2001).

Despite federal regulations on child-directed marketing, the most recent report from the Federal Trade Commission shows food and beverage companies invested an estimated $1.8 billion between 2006 and 2009 to promote their products to children and adolescents (Powell et al., 2013). In stark contrast, in 2024, Team Nutrition, a United States Department of Agriculture (USDA) initiative that supports school nutrition programs, received only $8.6 million to provide training, technical assistance, and nutrition and physical activity education to schools and communities, including any marketing of healthy foods (FY 2024 Team Nutrition Training Grants for Meal Pattern Modernization and Retention and Mentorship Opportunities / Food and Nutrition Service, n.d.; Team Nutrition / Food and Nutrition Service, n.d.). SNAP (Supplemental Nutrition Assistance Program)-Ed, the education program associated with SNAP, received only 0.4% of the total SNAP budget, or about $5.15 annually for each of the nearly 90 million people utilizing the program, which must be divided between direct education and any fruit and vegetable promotion marketing efforts (Keller et al., 2024). While this funding was already low relative to industry marketing to children, the funding was discontinued altogether in 2025, further decreasing the exposure to healthy messages in schools.

While it is understood that child-directed marketing of unhealthy food products negatively impacts the diet quality of U.S. children (Scaglioni et al., 2018; Story & French, 2004), research is not as clear as to whether increasing the prevalence of healthful nutrition content could encourage children to make healthier food choices. It is possible that saturating the child’s environment with healthful nutrition marketing, as commercial food companies have for decades, may improve children’s dietary intake. Because the National School Lunch Program (NSLP) delivers lunches to over 30 million students daily (Billings, 2025), schools can be an advantageous setting to investigate the current marketing of foods to children to determine how to optimize marketing for healthful choices. Additionally, schools are advantageous because they can allow for isolated healthful food marketing, as schools are required to only market foods that meet certain nutrition standards, excluding the popular products of many food companies (Food and Beverage Marketing to Children, Adolescents, and Parents of Toddlers and Young Children Policy Statement, 2024). Few studies have examined which marketing approaches effectively encourage healthier food selection beyond simply increasing availability (Foerster et al., 1995; Lefebvre et al., 1999; McDermott et al., 2005; Siega-Riz et al., 2011).

Message framing is used in nutrition marketing to emphasize the advantages or disadvantages of certain dietary behaviors. Past research has found that framing the benefits of healthy eating in a positive light is typically most useful to improving attitudes and/or behavior (Binder et al., 2020b; Wyllie et al., 2015). Unhealthy food marketing typically focuses on pairing their product with a social or tangible benefits (e.g. free samples, and gifts), or celebrity influence (*WHO EMRO - Nutrition*, 2025) and highlights the product appeal, alongside strategic message framing. To our knowledge, no previous studies have analyzed the content of existing nutrition marketing designed to encourage children to engage in healthier eating behaviors in schools or if the use of nutrition-promoting strategies varies across grade levels or age groups.

The objective of this study is to examine the content of existing nutrition marketing materials used within elementary, middle, and high school cafeterias in Arizona to understand the current strategies used to promote nutrition concepts and to find patterns between school levels. This study is timely in addressing funding for nutrition promotion. Currently, resources for nutrition education are being defunded. In particular, SNAP-Ed had campaigns to promote fruit and vegetable consumption, such as The FNV Campaign (FNV, n.d.) and the Pick a Better Snack (Pick a Better Snack, n.d.) campaign. Now, SNAP-Ed is planned to have no further funding (Supplemental Nutrition Assistance Program Nutrition Education and Obesity Prevention Grant Program (SNAP-Ed) Questions and Answers / Food and Nutrition Service, n.d.). For this reason, understanding fruit and vegetable marketing is more important than ever to establish a foundation to evaluate the effectiveness of different strategies and identify gaps in school nutrition programming

## 2.1 Methods

### 2.2 Study Design and Sample

This research study included a content analysis of existing nutrition marketing within 37 school cafeterias in Arizona including elementary (n=13), middle (n=12), and high schools (n=12) between 2017–2020. The schools were recruited as a convenience sample from a separate study (Adams et al., 2019). Clinical trial number: not applicable. Human participant consent: not applicable.

Researchers captured photographs of the nutrition marketing materials during baseline data collection of student meal consumption for the primary study and during routine site visits before interventions. Site visits were conducted by trained student research assistants (RAs). To meet inclusion criteria, the materials photographed by the RAs were required to be a promotional material visible to the students within the cafeteria related to food and/or nutrition concepts. Nutrition concepts could be presented in either text or image form. The photos were coded by three coders and analyzed for their content, purpose, prevalence, visual messaging, and literary messaging used to influence eating behaviors in the school cafeteria.

### 2.3 Coding

Objective coding classifications were created for the present analysis to operationalize the coding procedure and create consistency among coders. Two coders reviewed the coding guidebook (seen in the appendix) and independently coded each photograph of the market material accordingly. Discrepancies were then discussed, and if an agreement could not be reached, a third coder made the final decision. The overall percent agreement between the three coders was strong at 93.1% ± 6.4% (kappa = 0.79 ± 0.16). The coding classifications were designed to capture content associated with the type of marketing material, purpose/content, messaging strategies, and literacy/language aspects of school cafeteria nutrition marketing. Definitions and examples of coding can be found in the appendix.

#### 2.3.1 Type of Marketing Material

The variables in the “type of marketing material” coding classification aimed to show what form of media was being used to deliver the nutrition message (e.g. flyers, posters, decals).

#### 2.3.2 Purpose and Content

The variables in the “purpose and content” coding classification aim to show what food products, behaviors and/or information were being promoted (e.g. fruit or vegetable behavior target, food group presence), what elements were used to attract the audience’s attention (e.g. cartoons, celebrity/athlete endorsement, social media/websites, emotional appeals, humor, brand appeal, etc.), and the frequency of these elements per material.

#### 2.3.3 Messaging Strategies

The variables in the “messaging strategies” coding classification aim to show the tone and structure of the messaging used to promote the product or behavior (e.g. message framing, message structure) and the time frame of any health messages present in the marketing in relation to the child audience (short vs. long-term health outcome message).

Message framing used the following four classifications: 1) gain-framed with positive reinforcement, 2) gain-framed with negative reinforcement, 3) loss-framed with negative punishment, and 4) loss-framed with positive punishment. Gain-framing is framing a message by highlighting the benefits that come from engaging in a behavior. Loss-framing is framing a message by highlighting what will be lost after performing a behavior. Positive reinforcement describes a positive outcome, such as eating fruits and vegetables can improve one’s mood, that occurs after a behavior. Negative reinforcement describes the escape from or prevention of a negative outcome that occurs from engaging in a behavior, such as eating more fruits and vegetables can reduce chances of getting sick. Negative punishment describes losing a desirable outcome, such as a fun activity, for engaging in a behavior. Certain fruit or vegetables may take too much time to eat and as a result a student would lose the opportunity to participate in fun physical recess activities. Positive punishment describes an undesirable stimulus that occurs following a behavior, such as eating too many fruits and vegetables can lead to unpleasant consequences, such as feeling sick or constipated.

#### 2.3.4 Literacy and Language Aspects

The content variables associated with the “literacy/language aspects” coding classification aim to show whether the reading level of the text incorporated in the marketing material was appropriate for the target grade according to Flesch-Kincaid scores and whether materials were available in different languages. The Flesch-Kincaid system provides two readability scores: the Flesch Reading Ease score, which ranges from 0 to 100 with higher scores meaning more readable text, and the Flesch-Kincaid Reading Grade score, which indicates the U.S. school grade level required to understand the text. Scores are derived from calculations based on average sentence length and the average number of syllables per word.

### 2.4 Statistical Analysis

Interrater reliability (kappa) was assessed to determine agreement between coders on each variable and then was averaged across each category. Descriptive statistics were run for each variable. To compare the prevalence of variables by grade level, a binary logistic regression was used to detect significant differences in the prevalence of content between elementary, middle, and high schools. A logistic regression was rerun on the sample, with elementary school as the referent category to detect whether the variables differed significantly in middle schools or high schools as compared to elementary schools. After applying a Bonferroni adjustment for the number of statistical tests run (n=41), significance was detected at or below a p-value of 0.001 An analysis of text-specific variables (e.g., message structure, message framing) was only run on the sample of nutrition marketing that contained text (total sample n=269, elementary n=108, middle n=80, high school n=81).

## 3.1 Results

All schools had at least one marketing material. After analyzing the sample of nutrition marketing photos for visual aspects, posters (40.1%) and flyers (25.0%) were found to be the most common types of nutrition marketing, and most were small (39.4%) or medium (37.3%) sized (table 1).

Table 2 shows nutrition marketing materials that encouraged taking a fruit or vegetable (47.5%) were more prevalent than materials that encouraged consuming a fruit or vegetable (28.2%). Otherwise, 40.1% of the marketing did not encourage taking or consuming a fruit or vegetable at all, with phrases such as “peppers have vitamins A, C, and potassium “ which, although still promoting a healthy food, does not directly encourage a behavior. Other pieces of marketing that were in this “neither” category simply had characters with food items without any messaging. The food group presented was most commonly fruit (68.3%), followed by vegetables (66.2%). Often, both fruit and vegetable marketing can occur within a single marketing material and result in a similar presentation rate. Dairy, grains, and meat or meat alternatives were also promoted at a lower rate.

The most common marketing strategies employed across the sample were references to social media/website (24.6%), which utilized social media or website links to engage with students (i.e., link to website, QR Code, reference to Facebook or Instagram), and the use of cartoon characters (16.5%) (table 2). Almost half of the nutrition marketing materials did not utilize any marketing strategy at all (49.6%), 27.3% utilized only one marketing strategy, and 22.1% utilized two or more strategies (table 2).

When the prevalence of marketing content in the sample was compared across grade level, marketing content such as social modeling, cartoon characters, and humor differed significantly by grade level (p<.00001), and the prevalence of cartoons differed significantly in middle schools compared to high schools (p<.00001) (table 2). The amount of marketing materials that featured one type of marketing content differed significantly across grade level (p<.00001), withsignificantly more marketing materials in high schools (p<.00001) (table 2).

Analysis of marketing aspects embedded in the nutrition marketing materials indicated that gain-frame message framing with positive reinforcement appeared most frequently in the sample of nutrition marketing with text (19.3%), followed by gain-framed with negative reinforcement (1.5%), loss-framed with negative punishment (1.1%), and loss-framed with positive punishment (0.4%) (table 3). The remaining marketing photos with text did not follow the textual structure of a gain or loss framed message (77.7%). Approximately 20% of the marketing contained health messages that mentioned short-term health outcomes (i.e., “eating fiber during lunch will help keep you full and full of energy through the day”) compared to 13% that contained health messages that mentioned long-term health outcomes (i.e., “drink milk for strong bone health”); 66.9% indicated no time frame.

The final content category investigated was the literacy and language aspects of the nutrition marketing sample. The average reading level of the nutrition marketing materials with text across the sample was a 5.1 grade level. Using the Flesch Kincaid readability tool, 16.7% of the marketing contained text was too high for the target grade level (Table 4). The majority (98.9%) of the nutrition marketing materials with text were written in English, and only 1.1% of the materials (n=3) were written in Spanish.

## 4.1 Discussion

The purpose of this content analysis was to build an understanding of what current nutrition marketing strategies are being used to promote healthy eating behaviors among children and adolescents in Arizona schools. The sample of nutrition marketing materials emphasized the behavior target of selecting fruits and vegetables (F/Vs) over the consumption of F/Vs. We found that the most common type of marketing in school cafeterias were flyers and most of the materials were small in size. About half (49.6%) of the materials in the sample did not use a specific marketing strategy (i.e., did not use celebrity endorsement, cartoons, humor, etc.), but the half that did utilize techniques used those that are known to be appealing to student demographics (Federal Trade Commission, 2012; Ohri-Vachaspati et al., 2015b; Story & French, 2004). This novel study is the first to code the content of nutrition marketing in school cafeterias and compare the prevalence of content across grade levels. While nutrition promotion materials are provided to school nutrition staff to put on display for their students, little is known about how these materials are designed to engage students. This study aimed to address the existing literature gap by initiating a conversation about determining current child nutrition marketing practices within the school cafeteria setting.

### 4.2 Comparison with Food and Beverage Marketing

Our findings can be interpreted within the context of the larger food marketing environment in which children are embedded. Children often choose food items recognized as less healthful, because of the overwhelming exposure to food and beverage marketing that is deliberately designed to be enticing (Federal Trade Comission, 2012). Compared to the relatively limited and inconsistent implementation of nutrition marketing in schools, food and beverage companies invest billions of dollars annually into multi-platform strategies, including television, social media, digital games, restaurant promotions, and even in-school marketing, to establish brand recognition and influence food preferences (Calvert, 2008; Mehta et al., 2012; Ohri-Vachaspati et al., 2015a; Powell et al., 2013). Research shows that corporate marketing materials targeting children are often large, visually stimulating, and strategically placed for maximum visibility (Mehta et al., 2012; Ohri-Vachaspati et al., 2015a), but the schools in this sample were displaying smaller materials, mainly in the form of standard paper-sized posters, which could be less noticeable or engaging to students.

Child-oriented food marketing overwhelmingly promotes unhealthy products, typically foods high in sugar, sodium, and saturated fat, with many of these products still carrying misleading health claims (Mehta et al., 2012). The ability for large food and beverage companies to invest funds into effectively marketing unhealthy foods to children creates further imbalance, as previous research indicates that both students and school nutrition professionals find nutrition marketing within schools not persuasive or compelling (Bruening & Adams, 2023). Nutrition marketing materials from the sample schools mostly promoted fruits or vegetables, but also promoted all the food groups considered part of a healthy dietary pattern according to the Dietary Guidelines for Americans (Dietary Guidelines for Americans, 2020–2025, n.d.), including proteins and whole grains. The promotion of fruits, vegetables, proteins, and whole grains mirrors many health campaigns, which often emphasize fruits and vegetables as the central focus of dietary change (FNV, n.d.; Foerster et al., 1995).

#### 4.2.1 Use of Cartoon Characters, Celebrities, and Social Media

The use of cartoon characters was among the most common marketing strategies and made up 16.5% of total marketing materials. Cartoons were the number one marketing strategy for elementary schools (31.5%) and second for middle schools (10.1%). In high schools, the use of cartoon characters was not as prevalent (4.6%). The common use of cartoon characters is notable as cartoon characters are regularly depicted to promote unhealthy food choices. Children who watch cartoons that show unhealthy foods are more likely to choose an unhealthy snack following the exposure compared to children who do not see any unhealthy food placements in cartoons (Naderer et al., 2018). As cartoon characters can be used to promote less healthy food for children, this strategy can be useful to promote healthy foods. Cartoons promoting fruits have been shown to lead children to increase their fruit intake (Binder et al., 2020a). For this reason, this marketing method can help optimize children’s food choices.

Celebrities were only used to promote healthy food choices in 5.3% of materials, much lower than cartoon characters, or social media and websites (24.6%). Celebrities can be a powerful influence on students. Celebrities in food advertising increase the intake of foods high in fat, salt, and sugar (Packer et al., 2022). This can be impactful as celebrities promote food and beverage products more than any other category of product, other than consumer goods (with 26% of promotions being consumer products and 18% of promotions being food and beverage products) (Bragg et al., 2016). Celebrity endorsements have been proven to increase the use or consumption of the promoted product (Primack et al., 2014), and up to 80% of the food and beverage products promoted are considered energy-dense and nutrient-poor (Bragg et al., 2016). Leveraging celebrities for healthier food promotion in schools could therefore represent an untapped strategy to counterbalance the dominant influence of unhealthy celebrity-endorsed products.

Marketing using social and website media links was the most common (24.6%). Social media marketing is growing rapidly. The social media influencer marketing size has grown from $1.7 billion per year in 2015 to $32.6 billion per year in 2025 (Global Influencer Market Size 2025, n.d.). Over 15% of the videos from the top 100 influencers in the US in 2025 contained a food or beverage product (Dupuis et al., 2025). Leveraging this growth by continuing to utilize social media as could be a tool for nutrition marketing in schools.

#### 4.2.2 Gain vs. Loss Message Framing

This study found that 22.3% of the school marketing sample used message framing. Gain-framing with positive reinforcement was most common (19.3% overall; 33.3% in high schools). Only 1.6% of the school marketing sample used loss-framing. Using gain-frame messaging is appropriate as gain-frame messages are typically more effective at encouraging ongoing healthy behaviors, while loss-framed messages are typically more useful for behavioral cessation or one-time behaviors (Binder et al., 2020b; Wyllie et al., 2015). Additionally, aligning message framing to student values can further strengthen impact (Bryan et al., 2016). Research has shown that student involvement in the design of marketing materials for fruits and vegetables increases fruit and vegetable consumption more than only being exposed to fruit and vegetable marketing materials (Gustafson et al., 2017). Including students in the co-design of school marketing may increase relevance, engagement, and ultimately impact on food choices. Effectiveness increases when messages are aligned with student values, such as linking healthy eating to social justice or climate action (e.g., “choose plant proteins to support climate-friendly eating”) (Bryan et al., 2016). Schools could benefit from marketing campaigns that use gain-frame messaging and integrate age-specific motivational values.

#### 4.2.3 Short-term vs. Long-term Messaging

Approximately 20% of the marketing contained health messages that gave short-term student health outcomes (i.e., “eating fiber during lunch will help keep you feel full and give you energy through the day”) compared to 13% that contained health messages that gave long-term student health outcomes (i.e., “drink milk to for strong bones”), while 66.9% indicated no time frame. The utility of short-term versus long-term framing is influenced by an individual’s self-view, with individuals who have an independent self-view responding more to long-term messages and individuals with an interdependent self-view responding more to short-term messaging (Pounders et al., 2015). This suggests that both short- and long-term health outcomes may be useful, but effectiveness may hinge on whether the message is framed around individual benefits or collective outcomes. Further research is needed to examine how message timeframes interact with child and adolescent self-identity to shape dietary behaviors.

### 4.3 Readability & Literacy

Elementary schools overall contained marketing materials written at a reading level higher than what would be appropriate for the targeted age group (grade 5.2). Marketing strategies are unlikely to be effective if all members of the intended audience cannot understand the materials. Similarly, 98.9% of the nutrition marketing materials with text were written in English, and only 1.1% (n = 3) were written in Spanish. Given that Spanish is the second most spoken language in Arizona, spoken in 20.4% of households, some students may be excluded from understanding key messages (The Demographic Statistical Atlas of the United States - Statistical Atlas, n.d.). Tailoring readability and language accessibility to diverse student populations could improve equity and reach of nutrition promotion.

### 4.4 Strengths and Study Limitations

The study is strong in that it uses first-hand data to categorize all marketing materials for each school in the sample. Additionally, there is little to no evidence on the topic of advertising content in child-directed nutrition marketing within school settings, nor on how the content compared across grade levels. This study photographed marketing materials within elementary, middle, and higher schools and rated multiple elements to capture the content, visual aspects, marketing strategies, and literacy/language aspects of the marketing within the sample. Additionally, the schools in this study were geographically diverse in urban and rural low-income schools across Arizona, which suggests that the results may be more valuable for underserved populations. The information provided by this study is valuable for developing future research for developing and refining FV marketing materials for school settings, which will be critical for continuing a dialogue that can foster the development of best practices for nutrition-based social marketing.

Limitations of this study include the use of a convenience sample of schools recruited by the primary study. A sample of nutrition marketing located in a larger, randomized sample of schools will allow for generalizability and stronger correlations between the presence of content variables across grade level. The Arizona school system has many K-8 and K-12 schools, but the data in this study did not include K-8 or K-12 distinctions in the analysis. In addition, the content analysis codebook was created for this study and, therefore, has not been validated as an assessment tool. The specific location within the cafeteria environment where the nutrition marketing was located was not recorded. This study does not draw any direct correlations or causation between content variables, and therefore, the impact of marketing materials on selection, consumption, and waste of fruits and vegetables is unknown. Lastly, the images of nutrition marketing in this study were taken at one time point, and therefore any changes or rotations of the materials over time were not detected.

### 4.5 Implications to Research and Practice

The information provided by this study offers key insights into the strategies used by K-12 schools to promote healthy food intake in school cafeterias. As children consume up to half of their daily intake in schools (CDC, 2025), schools are a pivotal method of influencing eating choices. The present study highlights how some schools have attempted to encourage healthy eating habits. While we see a variety of techniques, the utility of each method must be understood. This study can serve as a baseline for future researchers to investigate student responses to each marketing strategy. A notable finding is the discrepancy in the literacy level of materials used in elementary schools and the lack of Spanish materials. Schools are promoting healthy eating, but students may not understand the messages, negating the benefits.

The disparity between the intensity of unhealthy food marketing and the relatively limited reach of nutrition promotion in schools has important implications for child dietary behaviors and long-term health (Eskenazi & Enright, 2021; Hawkes, 2004; Quinn et al., 2018). While regulatory initiatives such as the Children’s Food and Beverage Advertising Initiative (CFBAI) have introduced stricter nutritional criteria for advertising, evidence suggests that many unhealthy products continue to dominate child-targeted advertising, including on children’s television networks (Wootan et al., 2019). In contrast, school-based nutrition promotion remains underfunded, fragmented, and often limited to small posters or flyers. A proportion of the extensive marketing budget from major food and beverage companies is used to research effective marketing strategies, which could prove valuable if applied to nutrition marketing as well. As prior research emphasizes, health campaigns and social marketing strategies can support dietary change, but their success depends on adequate visibility, the integration of evidence-based marketing principles, and sufficient funding (Carins & Rundle-Thiele, 2014; Evans, 2008; Pirani & Reizes, 2005). Without addressing the imbalance in exposure and fiscal investment between corporate and health-promoting marketing, nutrition messaging in schools will remain at a significant disadvantage.

## 5.1 Conclusion

The general understanding of the content and effects of nutrition marketing in school cafeterias on child and adolescent eating behaviors has yet to be built. This study provides insight into the content, strategy, literacy, and language aspects of a sample of nutrition marketing within elementary, middle, and high schools in Arizona. The study demonstrated a lack of implementation of marketing appeals. Further investment and research is needed in several areas of school nutrition marketing, including design, formative research processes, implementation effects, and long-term evaluation, to better compete with child-directed food and beverage marketing of unhealthy foods. As food companies can invest billions of dollars into promoting unhealthy food products, which are known to influence child eating habits, a substantial investment on creating and testing effective marketing methods for healthy foods in schools is needed.

## Supplementary Material

Supplementary Files

This is a list of supplementary files associated with this preprint. Click to download.

- Appendix.docx

## Figures and Tables

**Table 1:** The type of nutrition marketing materials (n=284) found in a sample of elementary, middle, and high schools in Arizona

	All Schools	Elementary	Middle	High School	p-value
Type of Marketing %(n)	(n=284)	(n=108)	(n=89)	(n=87)	
Poster	40.1% (114)	50.0% (54)	23.6% (21)^b^	44.8% (39)	0.3356
Flyer	25.0% (71)	24.1% (26)	30.3% (27)	20.7% (18)	0.648
Cafeteria Decoration	13.0% (37)	7.4% (8)	19.1% (17)	13.8% (12)	0.1527
Decals	9.2% (26)	11.1% (12)	7.9% (7)	8.0% (7)	0.4441
Banner	4.9% (14)	1.9% (2)	11.2% (10)	2.3% (2)	0.7312
Technological Display	3.5% (10)	0.0% (0)	1.1% (1)	10.3% (9)	0.0101
Table tent	2.5% (7)	5.6% (102)	1.1% (0)	0.0% (0)	0.0471
Sticker	1.8% (5)	0.0% (0)	5.6% (5)	0.0% (0)	0.8399

a statistically significant difference across grade levels (*p≤.001)

b statistically significant difference (*p≤.001) of middle and high schools compared to elementary schools (referent)

**Table 2:** The purpose^1^ and content of nutrition marketing materials (n=284) in a sample of elementary, middle, and high schools in Arizona

	All Schools	Elementary School	Middle School	High School	p-value
F/V Behavior Target %(n)	(n=284)	(n=108)	(n=89)	(n=87)	
Taking a F/V	47.5% (135)	41.7% (45)	48.3% (43)	54.0% (47)	0.0853
Consuming a F/V	28.2% (80)	34.3% (37)	11.2% (10)	37.9% (33)	0.7595
Neither	40.1% (114)	41.7% (45)	43.8% (39)	32.2% (28)	0.2003
Food Group Presence %(n)	(n=284)	(n=108)	(n=89)	(n=87)	
Fruit	68.3% (194)	72.2% (78)	66.3% (59)	65.5% (57)	0.3043
Vegetable	66.2% (188)	75.0% (81)	61.8% (55)	59.8% (52)	0.0224
Dairy	48.2% (137)	47.2% (51)	59.6% (53)	37.9% (33)	0.2580
Grain	45.1% (128)	42.6% (46	52.8% (47	40.2% (35)	0.8244
Meat/Meat Alternative (Protein)	40.1% (114)	35.2% (38)	44.9% (40)	41.4% (36)	0.3460
Marketing Content %(n)	(n=284)	(n=108)	(n=89)	(n=87)	
Social Media/Websites	24.6% (70)	25.9% (28)	10.1% (9)	37.9% (33)	0.0909
Cartoon Characters	16.5% (47)	31.5% (34)	10.1% (9)^b^	4.6% (4)^b^	0.0000^a^
Emotional Appeal	15.1% (43)	19.4% (21)	14.6% (13)	10.3% (9)	0.0794
Brand Appeal	7.7% (22)	10.2% (11)	6.7% (6)	5.7% (5)	0.2432
Interactivity	7.0% (20)	4.6% (5)	5.6% (5)	11.5% (10)	0.0743
Humor	7.0% (20)	14.8% (16)	4.5% (4)	0.0% (0)	0.0006^a^
Celebrity/Athlete endorsements	5.3% (15)	7.4% (8)	7.9% (7)	0.0% (0)	0.035
None	49.6% (141)	41.7% (45)	67.4% (60)	41.4% (36)	0.8375
One strategy	27.3% (79)	22.2% (24)	15.7% (14)	47.1% (41)^b^	0.0003^a^
Two strategies	14.2% (41)	21.3% (23)	9.0% (8)	11.5% (10)	0.044
Three strategies	5.5% (16)	10.2% (11)	5.6% (5)	0.0% (0)	0.0059
>Three strategies	2.4% (7)	4.6% (5)	2.2% (2)	0.0% (0)	0.0654

F/V = fruit and vegetable

[1] Purpose: content variables associated with the product or behavior being promoted, including how the message is promoted and who it is promoted by

a statistically significant difference across grade levels (*p≤0.001)

b statistically significant difference (*p≤0.001) compared to elementary school (referent)

**Table 3:** The messaging strategies^3^ present in nutrition marketing materials (n=284) in a sample of elementary, middle, and high school in Arizona

	All Schools	Elementary	Middle	High School	p-value
Message Framing %(n)	(n=269)	(n=108)	(n=80)	(n=81)	
Gain-framed, positive reinforcement	Gain-framed, positive reinforcement	19.3% (52)	16.7% (18)	8.8% (7)	33.3% (27)
Gain-framed, negative reinforcement	Gain-framed, negative reinforcement	1.5% (4)	0.0% (0)	0.0% (0)	4.9% (4)
Loss-framed, positive punishment	Loss-framed, negative punishment	1.1% (3)	2.8% (3)	0.0% (0)	0.0% (0)
Loss-framed, negative punishment	Loss-framed, positive punishment	0.4% (1)	0.0% (0)	0.0% (0)	1.2% (1)
Short-term v. Long-term Health Outcome Message %(n)	(n=269)	(n=108)	(n=80)	(n=81)	
Short-term	20.1% (54)	21.3% (23)	20.0% (16)	18.5% (15)	0.4633
Long-term	13.0% (35)	10.2% (11)	2.5% (2)	27.2% (22)	0.0039
Message Structure %(n)	(n=269)	(n=108)	(n=80)	(n=81)	
Slogan	43.0% (122)	46.3% (50)	31.3% (25)	58.0% (47)	0.3821
Encourage	39.4% (112)	38.9% (42)	31.3% (25)	55.6% (45)	0.0978
Command	22.7% (61)	16.7% (18)	38.8% (31)	14.8% (12)	0.7943
Remind	11.3% (32)	12.0% (13)	16.2% (13)	7.4% (6)	0.294

3 Marketing Strategies: content variables associated with the promotional techniques used to promote the product or behavior message which includes communication messages and marketing techniques

a statistically significant difference across grade levels (*p≤.001)

b statistically significant difference (*p≤.001) compared to elementary school (referent)

**Table 4:** The literacy and language aspects^4^ present in nutrition marketing materials (n=284) in a sample of elementary, middle, and high schools in Arizona

	All Schools	Elementary	Middle	High School	p-value
Reading Level Appropriateness %(n)	(n=269)	(n=108)	(n=80)	(n=81)	
Reading level not appropriate for target audience (too high for grade level)	16.7% (45)	37.0% (40)	3.8% (3)^b^	2.5% (2)^b^	0.0000^a^
Average Reading Level	(n=269)	(n=108)	(n=80)	(n=81)	
	5.1	5.2	4.6	4.7	
Language %(n)	(n=269)	(n=108)	(n=80)	(n=81)	
English	98.9% (266)	99.1% (107)	98.8% (79)	98.8% (80)	0.0362
Spanish	1.1% (3)	0.9% (1)	1.2% (1)	1.2% (1)	0.8761
Other	0.0% (0)	0.0% (0)	0.0% (0)	0.0% (0)	

4 Literacy and language aspects: content variables associated with appropriateness of the literacy/language of the text in the marketing material for the target age group and demographic

a statistically significant difference across grade levels (p≤.001)

b statistically significant difference (p≤.001) compared to elementary school (referent)

## Data Availability

The datasets used and analyzed during the current study are available from the corresponding author on reasonable request.
